# Influence of Process Parameters on the Resistivity of 3D Printed Electrically Conductive Structures

**DOI:** 10.3390/mi13081203

**Published:** 2022-07-28

**Authors:** Kacper Dembek, Bartłomiej Podsiadły, Marcin Słoma

**Affiliations:** Micro- and Nanotechnology Division, Institute of Metrology and Biomedical Engineering, Faculty of Mechatronics, Warsaw University of Technology, 02-525 Warsaw, Poland; kacper.dembek.stud@pw.edu.pl (K.D.); bartlomiej.podsiadly.dokt@pw.edu.pl (B.P.)

**Keywords:** additive manufacturing, fused deposition modeling, 3D printing, polymer matrix composite, conductive composites, structural electronics

## Abstract

With recent developments in conductive composites, new possibilities emerged for 3D printed conductive structures. Complementary to a vast number of publications on materials properties, here we investigate the influence of printing parameters on the resistance of 3D printed structures. The influence of printing temperature on the resistance is significant, with too low value (210 °C) leading to nozzle clogging, while increasing the temperature by 20 °C above the recommended printing settings decreases resistivity by 15%, but causing degradation of the polymer matrix. The limitations of the FDM technique, related to the dimension accuracy emerging from the layer-by-layer printing approach, greatly influence the samples’ cross-section, causing irregular resistivity values for different layer heights. For samples with layer thickness lower than 0.2 mm, regardless of the nozzle diameter (0.5–1 mm), high resistance is attributed to the quality of samples. But for a 1 mm nozzle, we observe stabilized values or resistance for 0.3 to 1 mm layer height. Comparing resistance values and layer height generated from the slicer software, we observe a direct correlation—for a larger height of the sample resistance value decrease. Presented modifications in printing parameters can affect the final resistance by 50%. Controlling several parameters simultaneously poses a great challenge for designing high-efficiency structural electronics.

## 1. Introduction

Additive manufacturing often called 3D printing, is a method used for production of 3D objects or finished parts by adding material, usually layer upon layer, contrary to the much more common subtractive techniques that rely on removing excess material to make a product. The process of manufacturing parts using 3D printing technology consists of several stages. In the first stage CAD (Computer Aided Design) software is used to create model data of the final part. Then, using CAM (Computer Aided Manufacturing) software, the model is divided into layers, print parameters are selected, and the printer head movements are programmed for each layer. Once the program is uploaded to the machine, the manufacturing process begins by depositing successive layers of material until the final part is obtained [1].

One of the leading 3D printing technologies is FDM (Fused Deposition Modeling) of FFF (Fused Filament Fabrication). It involves heating the filament feedstock, a material in the form of a thin wire, above its glass transition temperature and then extruding it through a special computer-controlled nozzle until a layer of the model is completed. The process is then repeated until all layers are deposited. FDM offers many advantages, including low cost of equipment and materials, printing speed, and simplicity of operation [1]. This manufacturing process uses heat to plasticise the material and thus, it relies on thermoplastic polymers such as ABS (acrylonitrile butadiene styrene), PLA (polylactic acid), and Nylon, with a relatively low melting point, allowing low-temperature heaters ranging from 200 °C to 250 °C. They usually offer medium mechanical strength compared to injection moulding [2], low thermal conductivity and are generally electrical insulators, with a resistivity of about 10^15^ Ωm [3]. Many previous and recent studies have investigated the effect of the process parameters and building orientation on the mechanical properties of the printed structures, as well as for single and multimaterial structures [4,5,6]. In order to improve their properties and increase the functionality of the FDM method, various additives are added to the polymers to modify the properties, creating composite materials.

Composites are materials consisting of two or more components with distinct properties. The majority material is called the matrix, and the minority is called the filler, but this might not be the case on rare occasions. As a continuous phase material, the matrix is responsible for giving shape to the structure, limiting the negative influence of environmental factors on the filler, and effectively transferring forces deep into the composite. The main task of the filler is to provide strength, stiffness, and other desired properties. The filler is a heterogeneous phase uniformly distributed in the matrix. Composites can be divided according to matrix materials, type and material of the filler, properties, and applications. For example, a distinction is made between polymer matrix composites, ceramic matrix composites, metal matrix composites and even cement matrix composites. The filling in composites can take different shapes, which can modify the properties of the composite. Most commonly, the filler takes the forms of continuous and discontinuous fibres and powders [7]. Composites are a group of materials used in virtually every aspect of our lives, including transport, construction, or electronics, due to their numerous advantages [8].

Of particular interest in the context of 3D printing are polymer matrix composites, in which thermoplastics such as PLA and ABS can be used as the matrix. Single-walled or multi-walled carbon nanotubes, graphene nanoplatelets or carbon black can then be used as a filler [9]. With variable filler content, it is possible to obtain a significant change in materials properties, such as increased mechanical strength, better thermal properties, higher electrical conductivity, and enhanced aesthetic qualities, depending on the type of filler and its content [10,11].

Recently, conductive composites have received an increase in attention in the context of 3D printing. Due to their unique properties and the possibility of producing electrically conductive structures using the FDM technique, new applications are emerging. This extends the use of 3D printing technology into the field of printed electronics [9]. However, it is still a relatively new technique that requires further research. Most of the work carried out in this direction focuses on the fabrication of conductive composites and the study of their properties. However, the influence of printing parameters on the resistance of structures is rarely addressed. Several previous works cover the influence on the resistivity of 3D printed paths from printing temperature, printing and travel speed, fill pattern, orientation and extrusion, with limited analysis of nozzle diameter and layer height [12,13,14]. They are often limited to at most two nozzle diameters or two selected layer thickness values, focused on other aspects of FDM deposition such as printing orientation, the embedding of paths, build platform temperature and printing speed. Therefore, our research covers a broader regime of nozzle diameters and layer height values, and the correlation between these printing parameters with their influence on the resistance of 3D printed structural conductive paths.

## 2. Materials and Methods

The filament used in this study is commercially available Ampere PLA (Print-me, Gorzów Wielkopolski, Poland), with PLA polymer as matrix and carbon nanotubes as the filler. The filament diameter is 1.75 mm, density 1.35 g/cm^3^, and Young modulus 1500 Mpa. According to the manufacturer, recommended printing settings for this material are [15]:

- Printing temperature: 220–230 °C,
- Build platform temperature: 40–60 °C,
- Cooling of the print: 0–30%,
- Extrusion: 100–105%,
- Minimal nozzle diameter: 0.4 mm,
- Printing speed: 20–30 mm/s.

The models were printed on an FDM 3D Graften Pro printer (Graften, Olsztyn, Poland) [16]. It is a two-head printer supporting nozzle sizes from 0.2 mm to 1 mm and layer thickness from 0.09 mm to 0.8 mm. The maximal nozzle temperature is 300 °C. It has a work area of 218 mm × 260 mm, a print height of 240 mm and a speed up to 250 mm/s.

**Figure 1:**
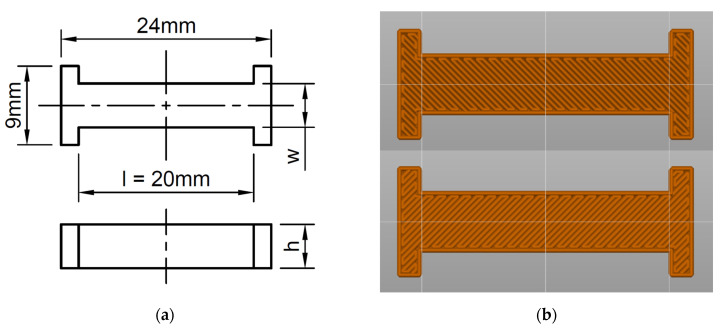
(**a**) Schematic drawing with dimensions of 3D printed samples for electrical measurements: top and side view. Dimensions of the samples: width (*w*) 5 mm, height (*h*) 5 mm, measurement length (*l*) 20 mm; (**b**) Image exported from the slicer software illustrating printing infill pattern for the sample layer *n* and *n* + 1 (45° and −45°, respectively).

The conductive beams were 3D printed from the filament at different print parameters to investigate the influence of printing parameters on resistivity. The shape and dimensions of a test sample are presented in Figure 1a. For each set of parameters, three beams were made for simplified statistical analysis. The resulting resistances were then converted to resistivity according to Equation (1):

(1) $$\rho =\frac{R\cdot w\cdot h}{l}$$

where:

*ρ*—Resistivity [Ωcm],

*R*—Resistance of the sample [Ω],

*w*—Width of the sample [cm],

*h*—Height of the sample [cm],

*l*—Length of the sample [cm]. 

The beams were printed with a 100% fill level and a linear fill pattern, with the path printing angle 45° and −45° to the direction of current flow (Figure 1b). This fill pattern compromises sufficient mechanical strength and the lowest possible resistance [17]. 

To minimise the negative effect of contact quality on the resistance measurement, a conductive silver paste was applied to the contact fields and dried overnight. Silver paste dried for a long time at room temperature was used because the composite filament is based on the PLA matrix, which is not resistant to high temperatures. According to the manufacturer’s recommendations, the material should not be used at temperatures above 60 °C, making it impossible to use standard conductive silver pastes cured at high temperatures (usually above 100 °C). With the silver paste applied, the resistance results were significantly lower than without, and the measurements were also highly repeatable. After the paste dried, resistance measurements were made using a Hameg HM 8112-3 precision multimeter. A double-T shape of the sample was initially designed to perform measurements with a four-point probe (extended ends of the samples were intended for such measurements). Initial measurements for both techniques, four-point and two-point, resulted in comparable resistance values. Therefore, two-point measurements were introduced for final measurements as less time-consuming. Figure 1a presents the design of the sample in two views: top and side.

## 3. Results and Discussion

### 3.1. Influence of the Printing Temperature on the Resistivity

**Figure 2:**
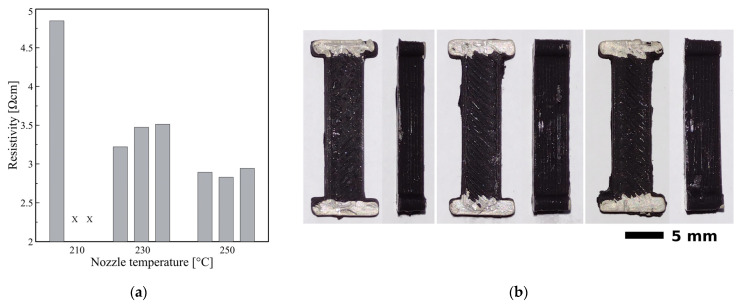
Results from the experiments concerning influence of the printing temperature on the quality and resistivity of the samples for temperature values 210 °C, 230 °C and 250 °C. (**a**) Resistivity values for beams printed with different nozzle temperatures—“X” mark inform about failure due to the nozzle clogging; (**b**) Images of sample beams printed with different nozzle temperatures (from left): 210 °C, 230 °C and 250 °C, respectively.

First experiments are focused on the influence of the printing temperature on the resistance of printed beams. For this purpose, samples were printed using a 0.6 mm diameter nozzle with a layer height of 0.2 mm at a constant printing speed equal to 20 mm/s and 100% extrusion. The nozzle temperature for samples was set to 210 °C, 230 °C and 250 °C, respectively. Results of the measurements and images of the samples are presented in Figure 2.

When the printing temperature was set to 210 °C, below the manufacturer’s recommended temperature, only one effective beam was obtained. However, the print quality of the obtained sample was unacceptable, while not all layers were printed correctly, and the beam was structurally poor. Also, the sample was significantly thinner than expected, which implicates the problems with the extrusion of a sufficient amount of material. During the next two attempts, the nozzle was clogged due to insufficient plasticisation of the material in the extruder. Therefore 210 °C temperature was excluded from further experiments. On the other hand, the printing process at 250 °C was flawless, and the beams were of good quality. The surface was smooth and uniform, and the layers seemed better connected. Unfortunately, at the same time, during the printing test at 250 °C there was a distinct odour of thermally degrading polymer during all printing attempts at this elevated temperature. Therefore this setting was also excluded from further experiments. At a print temperature of 230 °C, the manufacturer’s recommended temperature, the printing process was smooth, with no encountered problems.

We observe a distinct decrease in the resistivity value of samples printed in higher nozzle temperatures. When changing the temperature from 210 °C to 230 °C, the resistivity value dropped from 4.85 Ωcm to 3.40 Ωcm (29.8%). A further change of the nozzle temperature from 230 °C to 250 °C resulted in a decrease to 2.89 Ωcm (additional 15%).

### 3.2. Influence of the Layer Height and Nozzle Diameter on the Resistivity

The next step covered the influence on the resistance of samples from other important printing parameters: the layer height and the diameter of the extrusion nozzle. For this purpose, samples were printed with several different layer heights using different nozzle diameters. As a rule of thumb, a good practice for FDM printing is that the layer height should be close to 50% of the nozzle diameter and not exceed 80%. In this experiment, we use nozzles with diameters from 0.4 mm to 1 mm, so it is clear that not all layer height values will be covered for all nozzles diameters. All samples were printed at the temperature of 230 °C and printing speed of 20 mm/s at the extrusion rate of 100%.

**Figure 3:**
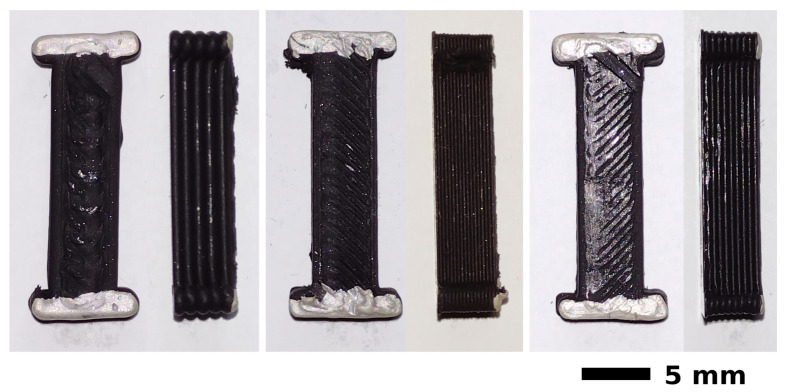
Representative samples of printed conductive beams (from left): 1 mm nozzle and 1 mm layer height; 0.5 mm nozzle and 0.3 mm layer height; 0.6 mm nozzle and 0.5 mm layer height.

The first tests with 0.4 mm and 0.5 mm nozzle diameter were very problematic and sometimes impossible. Samples printed with a 0.4 mm nozzle were defected, interrupted during the printing due to the nozzle clogging or not printed at all—this was the case for the layer heights 0.1 and 0.2 mm. Occasional samples exhibited resistance close to 50 Ω, around two times higher than for other obtained samples, printed with larger nozzle diameters. For a nozzle diameter of 0.5 mm, it was only possible to obtain samples at a layer height equal to or above 0.3 mm. Attempts to print at a layer height of 0.2 mm ended with the nozzle clogging, and the printing process was interrupted. Such problems occurred even though the minimum nozzle diameter recommended by the printed manufacturer for regular polymers is 0.4 mm. Deposition of the composite materials with a high content of the functional phase in the form of carbon nanotubes is more complex than polymer printing. High filler content increases the viscosity of the composite and negatively affects its MFI (Melt Flow Index). The decrease in MFI is due to the nucleation effect between PLA and CNT, which enhances intermolecular forces and increases the activation energy required for viscous flow. Composites with low MFI value are difficult to extrude, especially when using small diameter nozzles. Larger diameter nozzles than used for pure polymers are generally recommended. [10,18,19]. Because of these problems, further tests were performed for 0.5, 0.6 and 1 mm nozzles. Selected samples are presented in Figure 3.

**Figure 4:**
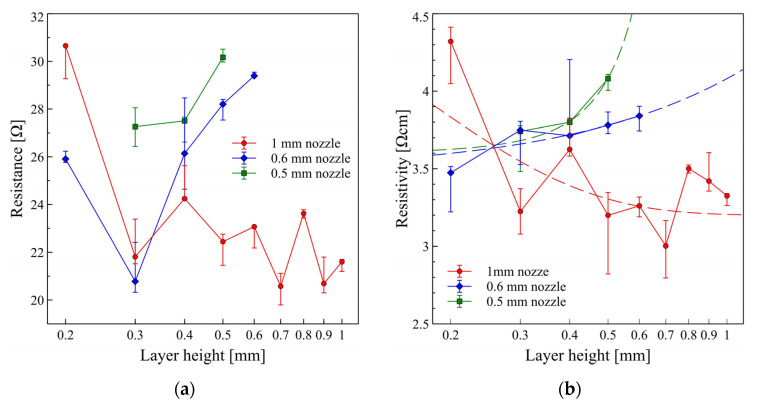
(**a**) Results of the resistance measurements of the samples printed with nozzle diameters from 0.5 to 1 mm, and wide range of layer thickness. (**b**) Calculated resistivity values for the same samples.

One of the main parameters considered during electrical and electronic circuits design is resistance of the paths and functional structures such as antennas or sensors. Printing process parameters might significantly influence final resistance of the paths directly affecting working parameters and efficiency of the electronic systems. Therefore, besides electrical resistivity being a materials property unrelated to the geometry of the elements, very useful information for the design of the circuits is from direct resistance measurements, presented in Figure 4a. Here we can observe two major effects: more stable and predictive resistance values can be obtained for paths printed with large nozzle diameters, and at the same time a significant resistance increase occurs when layer height is close to the value of nozzle diameter. This limits the use of small nozzle diameters, while it is problematic to print high viscosity polymer composites with small layer height (i.e., 0.1 or 0.2 mm). At the same time, there is a limited regime for large values of layer height (as a rule of thumb, it should not be more than 80% of nozzle diameter). Therefore, we could effectively print only 3 sample groups with a 0.5 mm diameter nozzle and only 5 sample groups with a 0.6 mm nozzle. For both of these nozzles, samples exhibit a visible increase in resistance. Only for a 1 mm nozzle we can obtain a broader process window regime for various layer heights. There is also an observable trend in resistance decrease, with a plateau from 0.5 to 1 mm layer height, but we have to keep in mind the fluctuations of dimensions related to the use of larger values of layer height. The observed high resistance values for samples with small layer thickness may be attributed to the lower printing quality of high viscosity composites, also observed in similar experiments covered in the literature [13].

The resistivity values are usually presented as the main factor when comparing the properties of different materials deposited with various techniques. While this parameter is generally geometry independent for bulk samples, it highly depends on the cross-section quality of the 3D printed samples, expressed as the uniformity of the printouts. For samples printed with 0.5 and 0.6 mm nozzle, we observe direct trends in resistivity change with layer height, while printing with layer thickness values close to the diameter of the nozzle is not advised, leading to less uniform printouts. For the samples printed with a 1 mm nozzle, offering a broader regime of layer thickness, the lowest resistivity values are attributed to the highest uniformity of printouts. Here more material is deposited in a single-layer print, minimizing the negative influence of parasitic resistances emerging from thin layers and contact resistance between them. Observed fluctuations for the 1 mm nozzle samples in the 0.3 to 1 mm layer thickness regime (plateau) result from the layer thickness applied to the slicer settings on the final sample height.

**Figure 5:**
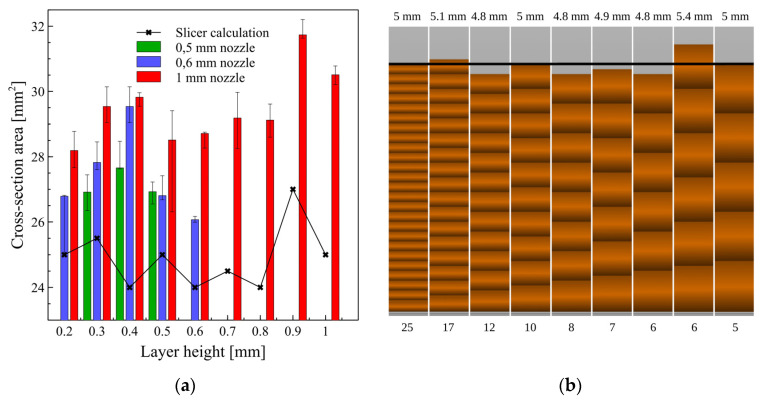
(**a**) Geometry measurements (cross-section areas) of the samples printed with nozzle diameters from 0.5 to 1 mm, and a wide layer thickness range. (**b**) Comparison of the samples generated by slicer with 5 mm height set as the initial value (black line)—upper values correspond to the calculated height as a multiplication of layer thickness, and bottom values correspond to the number of generated layers, respectively.

To have a more specific view of the influence of the most basic setting of the slicer, layer thickness, on the resistance of final layers, we need to analyse more closely the correlation between this parameter and the cross-section area of the samples. The summarized results of the geometry measurements and visualization of the geometry generated by slicer software are presented in Figure 5. Here, we clearly see high fluctuations in the measured cross-section area of printed samples, thou they all have been printed from a 5 mm × 5 mm CAD design. In a broader view, this is partially related to the limitations of the FDM technique, for which dimension accuracy of the printed element is in the range of 0.1–0.3 mm. But the most important influence is from the layer-by-layer printing approach, where the final height of the component is the multiplication of layer height and the number of layers generated by the slicer. For instance, with a layer height of 0.6 mm, the final height of the sample will be 4.8 mm, for 0.7 mm–4.9 mm, and 0.9 mm–5.4 mm, respectively. Due to these limitations, both cross-section area and resistivity values are so irregular.

**Figure 6:**
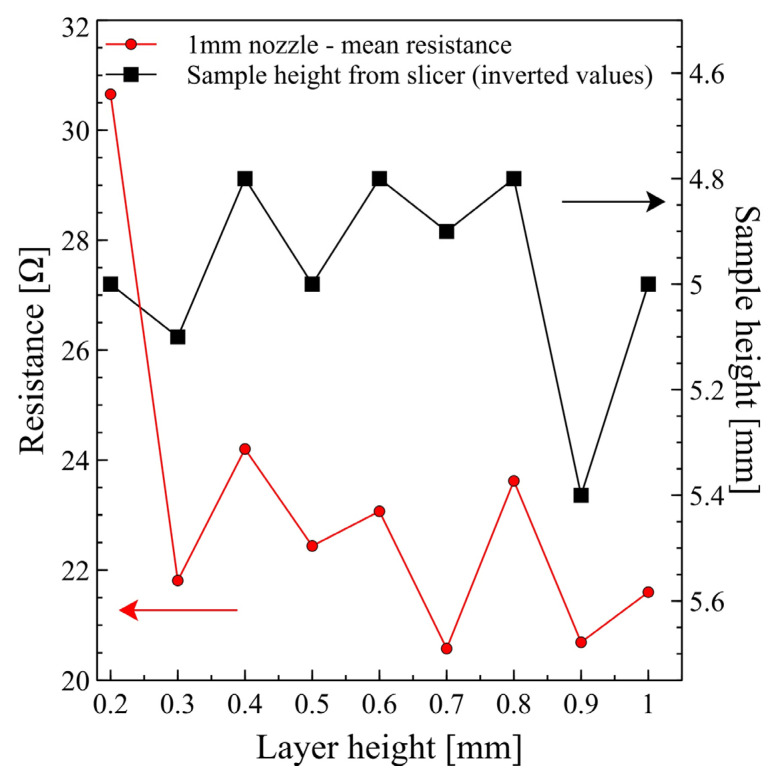
Mean resistance values for 1 mm nozzle printouts compared with sample height generated by slicer software—sample height plot intentionally inverted for better visualization of correlation.

Mentioned fluctuations in the sample cross-sections significantly influence printed samples’ resistance. Due to a large number of data for the 1 mm nozzle printouts, it is best to analyse such phenomenon only for these samples. Initially, we could attribute resistance fluctuations to the quality of the printed samples—simple and most common approach. This is true for samples with lower layer thickness, regardless of the nozzle diameter. As mentioned previously, for 0.5 mm nozzle diameter, it was only possible to obtain samples at a layer height equal to or above 0.3 mm—for 0.2 mm nozzle was clogged. Figure 4 shows similar negative results for 0.6 and 1 mm nozzle, with significantly high resistance values. Analysing results for a 1 mm nozzle more closely allows observing a trend in resistance change for roughly stabilized values obtained for 0.3 to 1 mm layer height. In Figure 6, a comparison of resistance values and sample height generated from the slicer software is presented—the sample height plot was intentionally inverted for better visualization of correlation. Keeping in mind that the resistance value will decrease with a larger sample height, we observe a direct correlation between these two values. Black colour points represent sample height values calculated by the slicer software as a multiplication of layer thickness and the number of generated layers. Besides results for 0.2 mm layer thickness, there is a straightforward correlation for remaining values of resistance and sample height—resistance decreases for thicker samples and resistance increases for thinner samples, respectively. This initially obvious observation that the resistance value drops with larger sample height (cross-section) is not so obvious taking into account that the designed geometry of the sample will be modified by the slicer without any information to the user and might significantly affect the working parameters and efficiency of the electronic systems.

### 3.3. Influence of the Extrusion Rate on the Resistivity

The other important process parameter influencing uniformity of the printed samples and thus the resistance is the extrusion rate, related to the volume of the material deposited during printing. Therefore, the influence of the extrusion parameter on the resistivity of the samples was also investigated. For this purpose, samples were printed using the most optimal settings observed in previous experiments, a 1 mm nozzle, layer height of 0.7 mm at 230 °C and print speed of 20 mm/s, changing only the extrusion parameter value. Three different values of the tested parameter were chosen: 95% (under extrusion), 100%, 105% (over extrusion). One sample was also printed at the extrusion value of 90%, but there were visible voids in the structure and poor adhesion of the layers, resulting in the degradation of sample properties and the highest resistance for measured samples (20.74 Ω). Results of resistance measurements and resistivity calculations are presented in Table 1.

Analyzing the results of the electrical measurements presented in Table 1, we can argue that for the samples printed with different values of extrusion rate, the uniformity of the samples varies, significantly affecting the final resistivity. Such phenomenon was also observed in other experiments presented in the literature and attributed to the higher extrusion pressure (filament-feeding force). Higher values of extrusion rate setting create higher shear rates in the nozzle, negatively affecting conductivity by breaking the conducting network in the composites [13]. This is important, while simple and most common measurements of the cross-section area based on the outside geometry might be insufficient. It is another essential aspect to consider when designing 3D printed structural circuits. Along with the results from the experiments with a 1 mm nozzle and different layer heights, this indicates that the most optimal effects with the lowest resistance values can be obtained when the final geometry of the conductive path (cross-section) is close to the diameter of the used nozzle.

## 4. Conclusions

This study investigates the effects of selected printing parameters on the resistance of 3D printed conductive composite structures. The parameters investigated included print temperature, layer height, nozzle diameter, and extrusion rate. The highest possible printing temperature, large nozzle size for composites, optimum layer height and extrusion values should be used to reduce the resistance of printed paths for structural electronics. Printing the samples at 250 °C instead of 230 °C led to a 15.13% decrease in resistivity but, at the same time, negatively influenced the polymer matrix. The lowest resistivity value (2.988 Ωcm) for 230 °C and 100% extrusion was achieved with a 1 mm nozzle and 0.7 mm layer height. Modifying the extrusion vales from 90% to 105% might affects the uniformity of the samples and effective cross-section, which is crucial for maximizing conductivity. In addition, difficulties were encountered in printing layers with 0.1 mm and 0.2 mm height with small diameter nozzles (0.4 mm and 0.5 mm) due to the clogging by composite material. Therefore, it is recommended to print composite structures with a minimum layer height of 0.3 mm and a minimum nozzle diameter of 0.5 mm to avoid clogging problems and achieve more uniform structures. The variations in the results show that a lot of attention needs to be focused on printing parameters besides the bulk conductivity of the composite, especially to maximize the values of such print quality-sensitive parameters like electrical conductivity. Future research should investigate additional parameters, such as printing speed, printing orientation, infill density, direction and type, or print build platform temperature, as well as check the investigated parameters over a broader range of values with more materials.

## Figures and Tables

**Table 1:** Resistance and resistivity values with geometry of the samples printed with different extrusion settings.

Extrusion [%]	Resistance [Ω]	Resistivity [Ωcm]	Cross-Section [mm^2^]
95	20.51 ± 0.75	2.87 ± 0.14	27.15 ± 0.21
100	20.49 ± 0.66	2.98 ± 0.18	29.13 ± 0.86
105	20.51 ± 0.96	3.13 ± 0.13	30.34 ± 0.11

## Data Availability

The data presented in this study are available on request from the corresponding author.
